# Risk factors for malaria deaths in Jalpaiguri district, West Bengal, India: evidence for further action

**DOI:** 10.1186/1475-2875-8-133

**Published:** 2009-06-16

**Authors:** Jagannath Sarkar, Manoj V Murhekar, Naman K Shah, Y van Hutin

**Affiliations:** 1 Field Epidemiology Training Programme (FETP), National Institute of Epidemiology (NIE), Indian Council of Medical Research (ICMR), R 127, Third Avenue, Tamil Nadu Housing Board, Phase I and II, Ayapakkam, Ambattur, Chennai, 600077, India; 2 School of Medicine, University of North Carolina, Chapel Hill, USA; 3 WHO India country office, New Delhi, India

## Abstract

**Background:**

In 2006, a cluster of malaria deaths in the highly endemic Jalpaiguri district, West Bengal, India, led to assignment of additional resources. Malaria deaths decreased, but continued to occur. A study was conducted to identify the risk factors for residual malaria deaths.

**Methods:**

Malaria death was defined as a death from fever with microscopically confirmed *Plasmodium falciparum *among residents of Jalpaiguri during 2007–2008. For each case, three age-, sex- and locality-matched controls were recruited among microscopically confirmed falciparum malaria patients cured during the same period. Clinical and treatment information was abstracted from records. Information about knowledge about malaria, presence of bed nets and DDT spraying was collected through interviews of the close relatives of study subjects. Odds ratio (OR) were calculated using multivariate methods.

**Results:**

51 malaria deaths were matched with 153 controls, which did not differ by age (median: 35 versus 36 years) and proportion of males (63% versus 63%). On multiple logistic regression analysis, compared with survivors, malaria deaths were more likely to have been admitted with already existing complications [OR = 4.1, 95% confidence interval (CI) = 1.6–10)], treated at a private facility (OR = 3.7, 95% CI = 1.2–12), received treatment after 48 hours of fever onset (OR = 14, 95% CI = 2.9–64), received chloroquine (OR = 13.3, 95% CI = 3.7–47). Households of the deceased were also more likely to miss bed nets (OR = 6.3, 95% CI = 1.9–24) and DDT spraying (OR = 9.2, 95% CI = 2.8–31).

**Conclusion:**

Elimination of malaria deaths will require education of providers for prompt referral before complications, engagement of the private sector, community awareness for early treatment as well as scaled-up use of bed nets use and DDT. Use of newer generation anti-malarials must to be generalized.

## Background

Effective malaria controls depends on early diagnosis and treatment, vector control and personal protection [1,2]. The objectives of a treatment policy are to ensure rapid cure, reduce morbidity and eliminate mortality through preventing the progression of uncomplicated malaria into severe, potentially fatal disease [1,3]. Chloroquine, used to be the mainstay of anti-malarial treatment. However, growing resistance led the World Health Organization to recommend that all uncomplicated *falciparum *infections be treated with an artemisinin-based combination therapy (ACT). Patients suffering from severe malaria presenting in primary health care facilities should be provided pre-referral treatment and transferred for full parenteral treatment and supportive care [2,3].

In 2007, the Indian National Vector-borne Disease Control Programme (NVBDCP) reported 1,502,742 malaria cases and 1,274 deaths [4]. However, cases and especially deaths may be under-reported. Reasons for under-reporting include management of two thirds of the patients by the private sector, absence of routine death certification in rural areas and poor infrastructure for malaria diagnosis in public health care facilities.

Jalpaiguri district, in the North of the West Bengal state, experiences perennial malaria transmission. During an outbreak in 2003, 842,409 clinical malaria cases and 149 deaths were reported. A 2003 drug resistance study reported 86% treatment failure to chloroquine among falciparum malaria cases. During a subsequent malaria outbreak in 2006, 62,020 confirmed malaria cases and 97 deaths were reported [Government of West Bengal, department of health, unpublished data]. During this outbreak, 40% of the cases and all malaria deaths were due to *Plasmodium falciparum *infection. Review of malaria control activities in the Alipurduar sub-division of the district (that reported 67 malaria deaths in 2006) pointed to inadequate human resources funds and logistics [5]. Deficiencies in the public health care system that normally managed malaria through case finding and treatment had led to a shift towards unqualified private practitioners, leading to poor management, which may have contributed to deaths [5]. The department of health took several measures to address deficiencies identified. It increased inputs for early diagnosis and treatment for patients attending public health facilities, vector control and personal protection measures: The number of malaria laboratory technicians in the district increased from 56 to 94 between 2006 and 2007 (an increase of 164%) and rapid diagnostic kits were provided for early diagnosis. Between 2006 and 2007, number of villages with drug treatment centres also increased from 899 to 1,284 (an increase of 70%). ACT (artesunate + sulphadoxine- pyrimethamine) coverage among falciparum patients attending public health facilities increased from 0% to 67%. In addition, the coverage of insecticide-impregnated bet nets among persons below the poverty lined increased from 0% to 21% during the same time period. However, the coverage of residual spraying with DDT decreased from 48% to 18% [Government of West Bengal, department of health, unpublished data]. Overall, the number of reported deaths reduced by 52% between 2006 and 2007, but they continued to be reported. Thus, a study was undertaken to identify the risk factors associated with a fatal outcome among patients with *falciparum *malaria (No deaths were reported among patient with *Plasmodium vivax *malaria).

## Methods

### Study population and design

A matched case-control study was conducted in Jalpaiguri district, West Bengal (Projected 2007 population: 3,973,930).

### Cases and controls

A malarial death case was defined as death in a febrile patient with microscopically-confirmed *P. falciparum *infection who resided in Jalpaiguri district between January 2007 and October 2008. Malaria deaths were recruited from the registers of the District Medical Office (DMO). Microscopically-confirmed falciparum malaria patients who were cured after treatment from January 2007 to October 2008 were considered as controls. To obtain these controls, surviving *P. falciparum *positive patients identified during the study period in different health care facilities were line-listed. From this sampling frame, three age- (± 5 years), sex- and locality-matched controls were selected for each malaria death case.

### Sample size

Assuming a prevalence of exposure among controls of 20% (i.e., treatment in the private sector), choosing a confidence interval (CI) of 95%, deciding for a power of 80% and opting for 3 controls per cases, the required sample size was 42 cases and 126 controls to detect an odds ratio of three. Anticipating a non-response of 10%, plans were made to recruit 47 cases and 141 controls.

### Data collection

Information about demographic and clinical characteristics as well as treatment details were abstracted from the 'malaria death investigation form' available for every malaria death case. For controls, demographic, clinical and treatment details were abstracted from hospital admission records and the malaria register. Information regarding knowledge, attitude, practices regarding malaria (including health-seeking behaviour during the last fever episode), personal protection and vector control measures was collected through interviews of close relatives of the death cases and controls. A pre-tested structured questionnaire was used to collect all information.

### Data analysis

The data analysis included calculation of age and sex-specific malaria death rates per 100,000 population using the projected population for 2007 as denominator and distribution of malaria deaths by month in 2007. With respect to health seeking behaviour, the study made two levels of comparison. First, it differentiated public health care facilities (sub-centres, primary health care centres, block primary health centres and hospitals) from private facilities (tea garden clinics and private hospitals). Second, it differentiated primary care (sub-centres, primary health care, block primary health centre in the public sector and tea garden clinics in the private sector) versus higher levels (public and private hospitals). A univariate analysis was used to calculate matched odds ratio (MOR) and their 95% confidence intervals (CI). After checking that there were no major differences between the crude and matched odds ratios in univariate analysis, variables with p value < 0.2 in univariate analysis and those of importance from a biological plausibility stand point were then included into a un-conditional logistic regression model as the conditional logistic regression model was unstable. All data analysis was conducted using Epi-info (version 3.5.1).

### Human subjects protection

The institutional ethics committee of the National Institute of Epidemiology, Chennai approved the study protocol. Written informed consent was obtained from the relatives of cases and controls before interviews.

## Results

### Descriptive epidemiology

In Jalpaiguri district, 51 malaria deaths were reported from January 2007 to October 2008 (Cumulated incidence of death over 22 months: 1.3 per 100,000). Malaria deaths were more common among males (1.5 per 100,000) and children under five years of age (2.3 per 100,000, Table 1). In 2007, malaria deaths occurred throughout the year, peaked during the rains, between May and July. They were reported from 11 out of 13 blocks. Blocks with high incidence of malaria deaths were remote, covered with tea plantations or forests, populated by poor people who often had to rely on the informal private sector or on private health care facilities in tea gardens.

**Table 1:** Cumulated malaria mortality incidence by age and sex, Jalpaiguri district, West Bengal, India, January 2007–October 2008

**Demographic characteristics**		**Malaria deaths**	**2007 population**	Mortality (per 100,000 population) *
Age group	< 5 year	10	431,992	2.3
	5–14 year	18	1,059,117	1.7
	15–29 year	11	1,065,175	1.0
	30–49 year	6	998,483	0.6
	50 year +	6	419,163	1.4

Sex	Male	31	2,046,504	1.5
	Female	20	1,927,436	1.0

**Total**		**51**	**3,973,930**	**1.3**

Cumulative rate for 22 months

### Analytic epidemiology

The 51 malaria death cases were matched with 153 controls. Cases and controls did not differ by age (Median: 35 versus 36 years) and proportion of males (63% versus 63%). Compared with survivors, malaria deaths were more likely to have been admitted with clinical manifestations of severe malaria (defined as unconsciousness, convulsions, coma, respiratory distress, circulatory collapse, haemoglobinuria and jaundice [6], MOR = 5.7, 95% CI = 2.5–13), to have been treated in the private sector (i.e., Non-government health care facilities, often staffed with poorly qualified health care workers, MOR = 17, 95% CI, 5.1–56) and to have received anti-malarial treatment > 48 hours after fever onset (MOR = 19, 95% CI = 2.1–170). Malarial deaths were more likely to have received chloroquine (MOR = 6.4, 95% CI = 2.6–15) and to live in houses without bed nets (treated or untreated, MOR = 5.8, 95% CI = 2.4–14). Absence of visits by health workers (MOR = 8.6, 95% CI = 2.3–32) and absence of DDT spraying in the house were also significantly associated with malarial death (MOR = 2.6, 95% CI = 1.3–5.2, Table 2).

**Table 2:** Frequency of selected exposures among cases and controls, Jalpaiguri district, West Bengal, India, 2007–8

**Exposure variables**		**Cases (n = 51)**		**Controls (n = 153)**		**MOR**	**95% CI**
				
		**#**	**%**	**#**	**%**		
Socio-demographic characteristics	Living below poverty level	49	92	147	97	0.83	0.31–2.4
	Being illiterate*,	43	84	132	86	0.82	0.32–2.3
	Being in a daily worker family	24	47	59	39	1.7	0.84–4.3

Treatment	Complications on admission	36	90	77	52	5.7	2.5–13
	Treated at private facility	25	49	11	7	17	5.1–56
	Treatment started >48 hours	11	22	15	10	19	2.1–170
	Treated with chloroquine	44	86	73	48	6.4	2.6–15
	Treated in primary care	45	78	142	54	0.56	0.19–1.67

Prevention in the last 12 months	Household not sprayed with DDT	26	51	42	27	2.6	1.3–5.3
	No bed net	40	78	63	41	5.8	2.4–14
	No house visit by health workers	21	41	25	16	8.6	2.3–32

Knowledge	Mosquito bite can cause malaria	1	2	15	10	0.22	0.03–1.4
	Malaria is curable	2	4	5	3	1.1	0.22–6.1
	Malaria causes anaemia	29	57	91	59	0.94	0.42–2.4
	Fever may kill if not treated early	49	96	139	91	0.53	0.14–2.3
	Blood test necessary when fever	2	4	10	6	0.83	0.31–2.4
	Malaria affects children	3	6	27	18	0.82	0.32–2.3
	Malaria affects pregnant women	3	6	2	1	1.7	0.84–4.3
	ITN prevent malaria	5	10	10	6	5.7	2.5–13

Confidence interval

*For young children, the literacy status of the head of family was considered

*Defined as the incapacity to read and write in the local language

*Fever onset

*Knowledge of the case-patients (deceased) and control subjects (survivors) assessed indirectly by proxy interviews of family members in both situations to ensure comparability of the data collection method

*Insecticide-impregnated mosquito nets

Variables significantly associated with malarial deaths in unconditional logistic regression model included presence of complications already upon admission (adjusted odds ratio [AOR] = 4.1, 95% CI = 1.6–10), management in the private sector (AOR = 3.7, 95% CI = 1.2 – 12), delayed anti-malaria treatment (AOR = 14, 95% CI = 2.9–64), use of chloroquine (AOR = 13, 95% CI = 3.7–47), absence of bed nets in the house (AOR = 6.3, 95% CI = 1.9–24) and absence of DDT spraying in the household (AOR = 9.2, 95% CI = 2.9–31, Table 3.)

**Table 3:** Exposures associated with malarial death in unconditional logistic regression, Jalpaiguri district, West Bengal, India, 2007–8

**Characteristics**	**Crude odds ratio**	**Adjusted odds Ratio**	**95% confidence interval**
Complications on admission	5.7	4.1	1.6–10
Treated at private facility	17	3.7	1.2–12
Treatment started >48 hours of fever onset	19	14	2.9–64
Treated with chloroquine	6.4	13	3.7–47
Household not equipped with bed nets	5.8	6.3	1.9–24
Household not sprayed with DDT	2.6	9.2	2.8–31

## Discussion

Patients diagnosed with malaria, should be treated early with safe and effective anti-malarial drugs, preferably within 24 hours of the onset of symptoms [6]. Inadequate and delayed treatment of uncomplicated malaria, especially in the non-immune patient may result in progression to severe malaria, which is associated with a high case fatality rate. In India, delay in treatment as well as presence of complications on admission were found to be key determinants of mortality [7-9].

In Jalpaiguri, delay in starting the anti-malarial treatment 48 hours after fever onset was significantly associated with death among falciparum malaria patients. This delay may be due to a combination of patient behaviour and health care access/quality issues. The association between presence of complications of malaria on admission and malaria death also suggests a delay in starting treatment.

Use of the appropriate anti-malarial drug is a key pre-requisite for malaria control and mortality reduction [2,6]. Combination therapies are preferred as they delay the emergence or spread of resistance [1,6]. ACT is highly efficacious and recommended in endemic regions. Considering the past history of chloroquine resistance in the district [10] and malarial deaths in 2006, ACT was made available in the block primary health centres. However, the results of the present study suggest that its use is still insufficient (almost half of control-subjects in the study received chloroquine) and that the failure to generalize this new treatment protocol contributed to the persistence of deaths.

As much as 30% of the population in the district resided in tea gardens and received treatment from private clinics there [5]. Malaria death was implicated with receiving treatment from private facilities in a previous study in Orissa [7]. In 2006, a survey of practitioners in private facilities in Jalpaiguri indicated that knowledge of malaria treatment and dangers signs was sub-optimal and lower than what was found in the public sector [5]. It is, therefore, necessary to educate the private providers in Jalpaiguri about the need to use the right anti-malarial drugs. This would not only reduce the case fatality but would also prevent development of resistance. Further professional training must also address early referral in the case of danger signs.

Not having at least one bed net in the house was associated with malaria death in this study. Use of bed nets is an important measure to prevent malaria and is one of the strategies of World Health Organization's Roll Back Malaria programme [2]. Insecticide-treated nets can reduce deaths in children by one fifth and episodes of malaria by half [11]. However, since we compared malaria deaths with malaria survivors, the reasons for the protective effect of bed nets are unclear. One possibility could be that bed nets reduced the intensity of infection. The other possibility could be that ownership of bed net was a surrogate marker of socio-economic status, through residual confounding. Only two variables were examined reflecting income: being the poverty line and belonging to the family of a daily labourer. Following the 2006 cluster of deaths, insecticide-treated bed nets were distributed. However, in 2007, in high-risk areas, only 5% of the population and 21% of the below poverty line families were covered. Furthermore, the proportion of houses coverage of DDT spraying activities decreased from 48% in 2006 to 18% in 2007. This failure to spray was also a risk factor for deaths in this study.

This study had a number of limitations. First, the information about clinical and treatment details from cases and controls was collected from available records instead of prospectively. Second, the study only included malaria deaths reported to the district medical officer. Malaria deaths occurring in the private sector were probably less likely to be reported than those occurring in the public sector. Thus, the study may have underestimated the proportion of death cases from the private sector. This limitation would have led to an under-estimation of the true odds ratio and does not affect the capacity of the study to conclude. Third, it is possible that respondents' knowledge about malaria and information about DDT spraying might have been influenced by the clinical outcome of study subjects. Fourth, presence of at least one bed net was considered as a proxy for use of bed nets in the house, as information about exact bed nets coverage in households and use was not collected during the study.

In conclusion, malaria deaths continue to occur in Jalpaiguri district despite additional investments in the anti-malaria programme. Based on these findings, a number of recommendations could be proposed. First, the Department of Health needs to sustain the inputs provided in the district in terms of rapid diagnostic kits, ACT and funds. It is also necessary to reach sufficient coverage (80%) of bed nets through more distribution, particularly to families living below the poverty line. Second, medical officers in primary care facilities need to be educated about early referral of malaria cases presenting with complications. Third, programme managers need to generate community awareness about malaria, including the need to seek prompt treatment for fever. Fourth, spray teams must catch up with the setbacks observed in terms of DDT spraying, particularly in high-risk areas. Fifth, private practitioners must be educated about use of appropriate anti-malarials, management of severe malaria and early referral. Specifically, to address issues in the private sector, an intervention based upon a pilot public-private partnership with tea plantations is now being considered in the district. This intervention would include additional training to improve curative and preventative malaria control, and an agreement by which ACT and bed nets could be supplied to tea garden industry for better malaria case management and prevention of transmission. Implementation of these measures must be monitored, including impact assessments using surveillance data, so that malaria deaths in Jalpaiguri can be eliminated.

## Conflict of interests

The authors declare that they have no competing interests.

## Authors' contributions

JS collected and analyzed the data, and drafted the manuscript. MVM participated in the study design, performed data analysis and drafted the manuscript. YH made substantial contributions to conception and design of the study. NKS and YH revised the manuscript critically for intellectual content. All authors read and approved the final manuscript.
